# Chronic blue light leads to accelerated aging in Drosophila by impairing energy metabolism and neurotransmitter levels

**DOI:** 10.3389/fragi.2022.983373

**Published:** 2022-08-31

**Authors:** Jun Yang, Yujuan Song, Alexander D. Law, Conner J. Rogan, Kelsey Shimoda, Danijel Djukovic, Jeffrey C. Anderson, Doris Kretzschmar, David A. Hendrix, Jadwiga M. Giebultowicz

**Affiliations:** ^1^ Department of Biochemistry and Biophysics, Oregon State University, Corvallis, OR, United States; ^2^ Department of Integrative Biology, Oregon State University, Corvallis, OR, United States; ^3^ Oregon Institute of Occupational Health Sciences, Oregon Health and Science University, Portland, OR, United States; ^4^ Department of Botany and Plant Pathology, Oregon State University, Corvallis, OR, United States; ^5^ The Northwest Metabolomics Research Center, University of Washington Medicine, Seattle, WA, United States; ^6^ School of Electrical Engineering and Computer Science, Oregon State University, Corvallis, OR, United States

**Keywords:** Drosophila, blue light, neurodegeneration, neurotransmitter, succinate, glutamate, energy metabolism, metabolomics

## Abstract

Blue light (BL) is becoming increasingly prevalent in artificial illumination, raising concerns about its potential health hazard to humans. In fact, there is evidence suggesting that acute BL exposure may lead to oxidative stress and death of retinal cells specialized for photoreception. On the other hand, recent studies in *Drosophila melanogaster* demonstrated that chronic BL exposure across lifespan leads to accelerated aging manifested in reduced lifespan and brain neurodegeneration even in flies with genetically ablated eyes, suggesting that BL can damage cells and tissues not specialized for light perception. At the physiological level, BL exposure impairs mitochondria function in flies, but the metabolic underpinnings of these effects have not been studied. Here, we investigated effects of chronic BL on metabolic pathways in heads of *eyes absent* (*eya* ^*2*^) mutant flies in order to focus on extra-retinal tissues. We compared metabolomic profiles in flies kept for 10 or 14 days in constant BL or constant darkness, using LC-MS and GC-MS. Data analysis revealed significant alterations in the levels of several metabolites suggesting that critical cellular pathways are impacted in BL-exposed flies. In particular, dramatic metabolic rearrangements are observed in heads of flies kept in BL for 14 days, including highly elevated levels of succinate but reduced levels of pyruvate and citrate, suggesting impairments in energy production. These flies also show onset of neurodegeneration and our analysis detected significantly reduced levels of several neurotransmitters including glutamate and Gamma-aminobutyric acid (GABA), suggesting that BL disrupts brain homeostasis. Taken together, these data provide novel insights into the mechanisms by which BL interferes with vital metabolic pathways that are conserved between fly and human cells.

## Introduction

Blue light (BL), which is characterized by high-energy short-wave light, has attracted interest as a potential health hazard to humans (Ouyang et al., 2020). BL is common in artificial lighting such as light-emitting diodes (LED) (Tsao et al., 2010; Lee et al., 2018) to which humans are increasingly exposed. There is increasing evidence that BL has the potential to damage human eyes contributing to diseases ranging from glaucoma (Ouyang et al., 2020) to retinal degeneration and age-related maculopathy (Algvere et al., 2006); however, little is known about the mechanisms of damage. Recent research on BL effects focused on human retina-related cells *in vitro* and suggest that BL can increase reactive oxygen species (ROS), cause DNA damage, impair mitochondrial function, and damage lysosomes (Ouyang et al., 2020; Kam et al., 2021). An *in vivo* model of acute BL phototoxicity was developed in the fruit fly, *Drosophila melanogaster*. It was reported that BL exposure induces phototransduction-dependent oxidative stress, lipid peroxidation and retinal degeneration in compound eyes (Chen et al., 2017).

Recent studies suggest that BL may be damaging to cells not specialized for phototransduction with such damage being reflected at the organismal level. For example, visible light, especially in the blue region, causes oxidative stress and shortens the lifespan of the nematode *C. elegans* (De Magalhaes Filho et al., 2018). Further, adult *Drosophila* exposed to 12 h of BL per day show symptoms of accelerated aging including impaired locomotor performance, brain neurodegeneration, and reduced lifespan compared to flies reared in constant darkness or LED light with blue wavelengths filtered out (Nash et al., 2019). These phenotypes were not only observed in wild type flies, but also in mutants with genetically ablated eyes (*eya* ^*2*^) kept in BL, suggesting that detrimental effects of BL, did not depend on the photoreceptor damage (Nash et al., 2019). Studies of mitochondria in the heads of *eya* ^*2*^ flies exposed to constant BL have connected the reduced lifespan to impairment of mitochondrial respiratory function, namely, reduced activity of Complex II in the electron transport chain (Song et al., 2022). It was also reported that sensitivity to BL is strongly age-dependent: BL reduces survival and significantly increases neurodegeneration in aged flies due to light-independent impairments in energy metabolism with age (Song et al., 2022).

Given that impairment of mitochondrial respiratory function by BL can affect brain function and lifespan in flies, it is important to understand how BL exposure affects metabolome composition. This question has not been addressed in any organism *in vivo* or *in vitro*. To understand how BL affects metabolic pathways in flies, we compared metabolomic profiles in heads of *eya* ^*2*^ flies kept in constant BL or constant darkness (DD). Our rationale for using constant light condition was based on recent reports that several metabolites show daily fluctuations in their levels that are regulated by the circadian clock and light/dark cycles (Rhoades et al., 2018). Constant light abolishes these fluctuations by stopping the clock. We have previously shown that genetic disruption of the clock does not alter lifespan of flies exposed to phototoxic effects of BL (Nash et al., 2019).

In this study, we used LC-MS and GC-MS to analyze changes in metabolite levels in flies exposed to constant BL before and after onset of BL-induced neurodegeneration. We report that the accelerated aging in *Drosophila* kept in BL is associated with significant changes in pathways involved in energy and amino acid metabolism and with altered levels of several brain neurotransmitters. The results provide new insights into the specific processes that are impaired by blue light in cells and tissues not specialized in phototransduction.

## Materials and methods

### Drosophila rearing, light exposure, longevity and neurodegeneration

*D. melanogaster* was maintained on a standard diet containing yeast (35 g/L), cornmeal (50 g/L), and molasses (5%) at 25 ± 1°C. Eyes absent (*eya* ^*2*^) mutants (Bloomington Drosophila Stock Center stock # 2285), which do not develop compound eyes (Bonini et al., 1993) were used in all experiments. Fly colonies were reared in cycles of 12 h of fluorescent light alternating with 12 h darkness. Flies used in the experiments were mated and separated by sex when 2–3 days old. Experimental adult males were maintained in constant darkness (DD) or constant blue light (BL) with a peak emission of 460 nm produced by the MarsAqua Dimmable 165W LED Light with a photon flux density of 20–30 μmol/m^2^/sec (irradiance of∼0.4 mW/cm^2^) measured at the level of horizontally placed narrow vials (Genesee Scientific), each containing 25 flies as previously described (Nash et al., 2019).

For each light condition, lifespan was measured using at least 50 males held in groups of 25 with mortality recorded and fresh diet provided every 2–3 days. Mortality curves were statistically analyzed using the log-rank test in GraphPad Prism 6.

To quantify BL-induced neurodegeneration in the brain, we measured the area of all vacuoles seen on sections of the brain as described previously (Carmine-Simmen et al., 2009). Analyses were done double-blind and statistical significance was determined with unpaired t-tests using GraphPad Prism 6.

### Succinate Dehydrogenase Assay

SDH activity was measured using the Succinate Dehydrogenase Activity Colorimetric Assay Kit (Sigma-Aldrich) as described (Song et al., 2022). Briefly, heads isolated from BL or DD males were homogenized with 100 µl of ice-cold SDH assay buffer, kept on ice for 10 min, and centrifuged at 10,000 × *g* for 5 min at 4°C. The supernatant (10 µl) was mixed with 92 µl of reaction mix (88 µl of SDH assay buffer, 2 µl of SDH substrate, and 2 µl of SDH probe) in each well and the absorbance was immediately read at 600 nm in kinetic mode for 30 min at 25°C using a BioTek Synergy 2 microplate reader. The SDH activity was calculated according to the manufacturer’s instructions and normalized to total protein concentration of the sample.

### ATP Assay

ATP was measured using the ATPlite Luminescence ATP Detection Assay System (Perkin Elmer) as described (Song et al., 2022). For each sample, 25 male heads were homogenized and the samples were centrifuged at 1,000 × *g* for 10 min at 4°C. For each sample, 60 µl of mitochondria-enriched supernatant was aliquoted to a fresh tube and boiled immediately for 10 min, then centrifuged at 20,000 × *g* for 5 min at 4°C. The steady state ATP content was measured in the supernatant in a white 96-well plate. 10 µl of supernatant was mixed with 90 µl of Schneider’s medium (Gibco, 21720-024) and 50 µl Cell Lysis Solution in each well. Then 50 µl of substrate solution was added to each sample well and mixed by shaking the plate for 5 min on an orbital shaker. The plate was dark adapted for 10 min and the luminescence was measured at 25°C using a BioTek Synergy 2 microplate reader. The ATP level was calculated using ATP standard curve and normalized to protein concentration of the sample.

### Liquid chromatography—Mass spectrometry

Males that were 2–4 days old were placed in BL or DD for 10 or 14 days, then collected for analysis. For each experimental condition, 8 samples of 50 fly heads each (7 samples for 14 BL conditions) were separated by vortexing flies in liquid nitrogen-cooled tubes. Aqueous metabolites for targeted LC-MS profiling were extracted as previously described (Kurup et al., 2021). Briefly, heads were transferred into bead tubes with ceramic 1.4 mm beads (Qiagen PowerBead Tubes) containing 200 µl purified deionized water at 4°C, then 800 µl of cold methanol was added (containing 124 µM 6C13-glucose and 25.9 µM 2C13- glutamate as internal standards to monitor sample prep). A pre-chilled Qiagen bead beater set at 30 Hz was used to homogenize tissue. The resulting homogenates were incubated at −20°C for 1 h, centrifuged at 20,000 × g for 5 min at 4°C, and 600 µl of the supernatant was transferred into a 1.5 ml microcentrifuge tube. Lastly, recovered supernatants were dried on a SpeedVac and reconstituted in 1.0 ml of LC- matching solvent containing 17.8 µM 2C13-tyrosine and 39.2 3C13-lactate (reference internal standards were added to the reconstituting solvent in order to monitor LC-MS performance). Samples were transferred into LC vials and placed into a temperature controlled autosampler for LC-MS analysis.

Targeted LC-MS metabolite analysis was performed on a duplex-LC-MS system composed of two Shimadzu UPLC pumps, CTC Analytics PAL HTC-xt temperature-controlled auto-sampler and AB Sciex 6500+ Triple Quadrupole MS equipped with ESI ionization source (2). UPLC pumps were connected to the auto-sampler in parallel and were able to perform two chromatography separations independently from each other. Each sample was injected twice on two identical analytical columns (Waters XBridge BEH Amide XP) performing separations in hydrophilic interaction liquid chromatography (HILIC) mode. While one column was performing separation and MS data acquisition in ESI+ ionization mode, the other column was getting equilibrated for sample injection, chromatography separation and MS data acquisition in ESI− mode. Each chromatography separation was 18 min (total analysis time per sample was 36 min). MS data acquisition was performed in multiple-reaction-monitoring (MRM) mode. LC-MS system was controlled using AB Sciex Analyst 1.6.3 software. Measured MS peaks were integrated using AB Sciex MultiQuant 3.0.3 software. The LC-MS assay was targeting 361 metabolites (plus 4 spiked reference internal standards). In addition, to the study samples, two sets of quality control (QC) samples were used to monitor the assay performance as well as data reproducibility. One QC [QC (I)] was a pooled human serum sample used to monitor system performance and the other QC [QC (S)] was pooled study samples and this QC was used to monitor data reproducibility. Each QC sample was injected per every 10 study samples. The data were well reproducible with a median CV of 4.6%.

### Gas chromatography—Mass spectrometry

GC-MS was performed on heads of males held in BL or DD for 14 days. Eight samples of 50 flies were used for each condition. For each sample, 50 male heads were separated by vortexing flies in liquid nitrogen-cooled tubes. Heads were transferred into bead tubes with ceramic 1.4 mm beads (Qiagen PowerBead Tubes). A Qiagen bead beater set at 30 Hz was used to homogenize tissue samples in 800 µl of pre-chilled (−20°C) 90% methanol containing 0.5 ng/ml ribitol as internal standard. The resulting homogenates were incubated at -20°C for 1 h, centrifuged at 20,000 × g for 5 min at 4°C, and 600 µl of the resulting supernatant transferred into a 1.5 ml microcentrifuge tube. The samples were evaporated to dryness in a centrifugal vacuum concentrator. The dried residue was resuspended in 10 µl of 30 mg/ml methoxyamine hydrochloride in pyridine (Sigma-Aldrich) and incubated at 37°C for 1.5 h shaking vigorously. Next, 25 µl of N-methyl-N-(trimethylsilyl) trifluoroacetamide with 1% trimethylchlorosilane (Covachem) was added and the samples were incubated at 37°C for 30 additional min with vigorously shaking. The samples were then transferred to glass autosampler vials, and 1 μl of the sample was injected with a 4:1 split into an Agilent 7890B GC system with a 30 m plus 10 m Duraguard × 0.25 mm × 0.25 μm DB-5MS + DG Agilent column. The oven temperature was kept at 60°C for 1 min, then ramped to 300°C at a rate of 10°C/min and held at 300°C for 10 min. Analytes were detected with an Agilent 5977B MSD in EI mode scanning from 50 m/z to 600 m/z. Mass spectrum analysis, component identification and peak area quantification were performed with AMDIS (Davies, 1998) using the FiehnLib (Kind et al., 2009) for automated component identification. A component was considered a match to a FiehnLib entry if the match score was >60 based upon spectral matching to the library entry, a retention time window of 6 s and strong retention-time drift penalties.

### Metabolite analysis

After removing any metabolites missing in more than 50% of samples, we were left with 175 LC 10-day metabolites, 176 LC 14-day metabolites, and 91 GC 14-day metabolites. Missing values in the remaining data were estimated with k-nearest neighbor and further normalized using “Statistical Analysis” function in MetaboAnalyst 5.0 web server (Pang et al., 2021). Data were normalized with sum normalization, log transformation, and auto scaling (computing z-scores) before *t*-test statistics (van den Berg et al., 2006). Density plots were created using the “seaborn” package of Python 2.7.14 (van Rossum, 1995; Hunter, 2007). Principal Component Analysis (PCA) was performed using the “factoextra” package of RStudio (Kassambara and Mundt, 2020; RStudio Team, 2020). Each PCA analysis was done between corresponding BL and DD group for the first five PC dimensions. PCA that minimize the intersection of the irregular polygon that contains all metabolites in each condition is shown in the figure with overlap rates. Overlap rates were computed using the “sf” package of RStudio (Opgen-Rhein et al., 2021). We plotted heatmaps to illustrate changes in metabolites after BL exposure using the “pheatmap” of package of RStudio (Kolde, 2019).

Metabolic pathway enrichment analysis and pathway topology analysis were conducted using the MetaboAnalyst 5.0 computational platform (Pang et al., 2021). Pathways were visualized using the “matplolib” package of Python 2.7.14 (Hunter, 2007). Geometric means between the BL and DD were calculated with the following equation: $\text{Geometric mean}=\;{\left(\overset{n}{\underset{i=1}{\prod }}p_{i}\right)}^{\frac{1}{n}}$ , where i represents metabolites belong to corresponding pathways, n represents the total metabolites belonging to that pathway, and $p_{i}$ represents the *p*-value for corresponding metabolites between BL and DD. Pathway impact and enrichment scores were computed using the MetaboAnalyst 5.0 (Pang et al., 2021).

Individual metabolites box plots were created with GraphPad Prism 6 and statistical significance was determined with unpaired *t*-tests.

## Results

### Effects of different duration of constant blue light on fly survival and neurodegeneration

**FIGURE 1:**
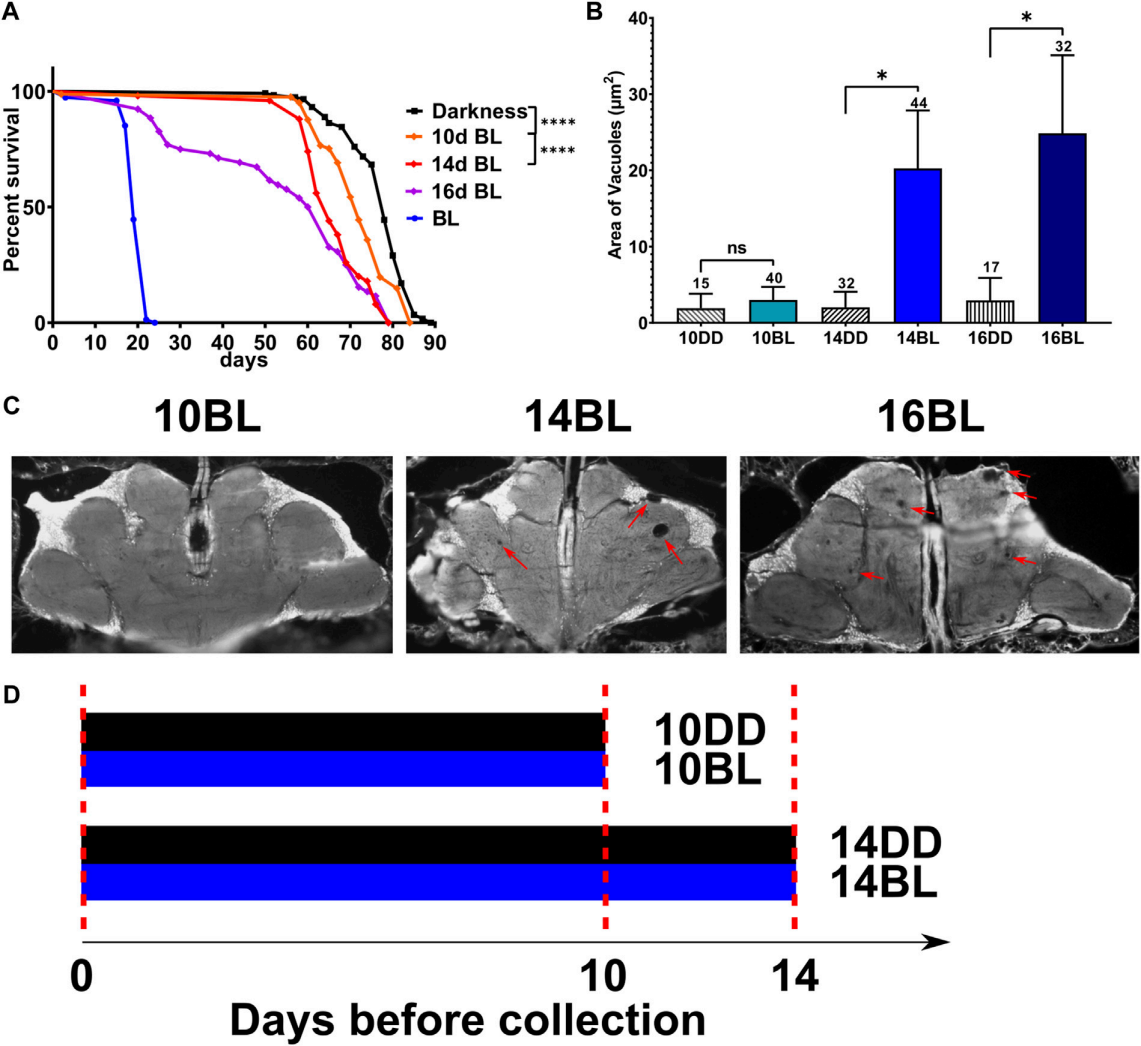
Effects of length of constant BL on lifespan and neurodegeneration in *eya* ^*2*^ flies. **(A)** Lifespan was measured in flies kept in constant darkness (DD), constant blue light (BL), or transferred from BL to DD after 10, 14, or 16 days in BL). Stars indicate significant differences in survival in BL based on statistics by log-rank test (^∗∗∗∗^ *p* < 0.0001). **(B)** Quantification of the average area of vacuoles in heads of flies exposed to BL for 10, 14, and 16 days and their age-matched DD control. Statistics by unpaired *t*-test (^∗^ *p* < 0.05) Numbers above bars indicate number of brains examined for each age and light condition. Error bars indicate standard error of the mean (SEM). **(C)** Representative images of brain sections showing brain vacuoles (red arrows) after indicated number of days in BL. **(D)** Scheme of the experimental design showing the treatment of flies collected for metabolomic profiles.

The longevity of *D. melanogaster* mutants with genetically ablated eyes (*eya* ^*2*^) is dramatically reduced in constant BL (Song et al., 2022). To investigate the timeline of damaging effects of BL, we tested the survival of eyeless male flies that were kept in constant BL for 10, 14, and 16-days and then transferred to DD. Mortality curves compared by log-rank test show that the survival of flies is significantly reduced proportionally to the increased number of days in BL compared to DD controls (Figure 1A). The lifespan showed a small but significant decrease in flies kept in BL for 10 days and was reduced more significantly in flies kept in constant BL for 14 days, but no flies died during BL exposure. However, among flies kept in BL for 16-days some died during and soon after BL exposure while others survived and continued living in DD (Figure 1A) showing that this cohort was composed of flies with reversible and irreversible BL damage.

We next evaluated brain neurodegeneration in *eya* ^*2*^ males exposed to constant BL for 10, 14, or 16 days compared to flies kept in DD for the same number of days (Figure 1B). For this purpose, heads were sectioned to measure the size of vacuoles indicative of brain cell loss. Flies kept in BL for 10d displayed negligible vacuolization similar to the DD control (Figure 1C). However, a significant increase in brain vacuolization was detected after 14 and 16 days of BL exposure compared to age-matched DD controls (Figure 1C). Given that BL did not affect brain vacuolization after 10 days of exposure but increased brain neurodegeneration in flies exposed to BL for 14 days, we investigated the effects of BL on metabolomic profiles at these two time points (experimental scheme in Figure 1D) to obtain insights into metabolite changes associated with the onset of brain damage.

### Metabolome of *Drosophila* heads changes with length of blue light exposure

We compared metabolome profiles in heads of males kept in BL or DD for 10 and 14 days using both liquid chromatography-mass spectrometry (LC-MS) and gas chromatography-mass spectrometry (GC-MS). Data from LC-MS or GC-MS were normalized through sum normalization, log transformation, and standard deviation scaling using MetaboAnalyst 5.0 (Pang et al., 2021) to limit sample loading difference and make all metabolites equally important irrespective of measurement errors (van den Berg et al., 2006). All metabolites detected in our study along with statistical analysis are shown in Supplementary Table S1.

**FIGURE 2:**
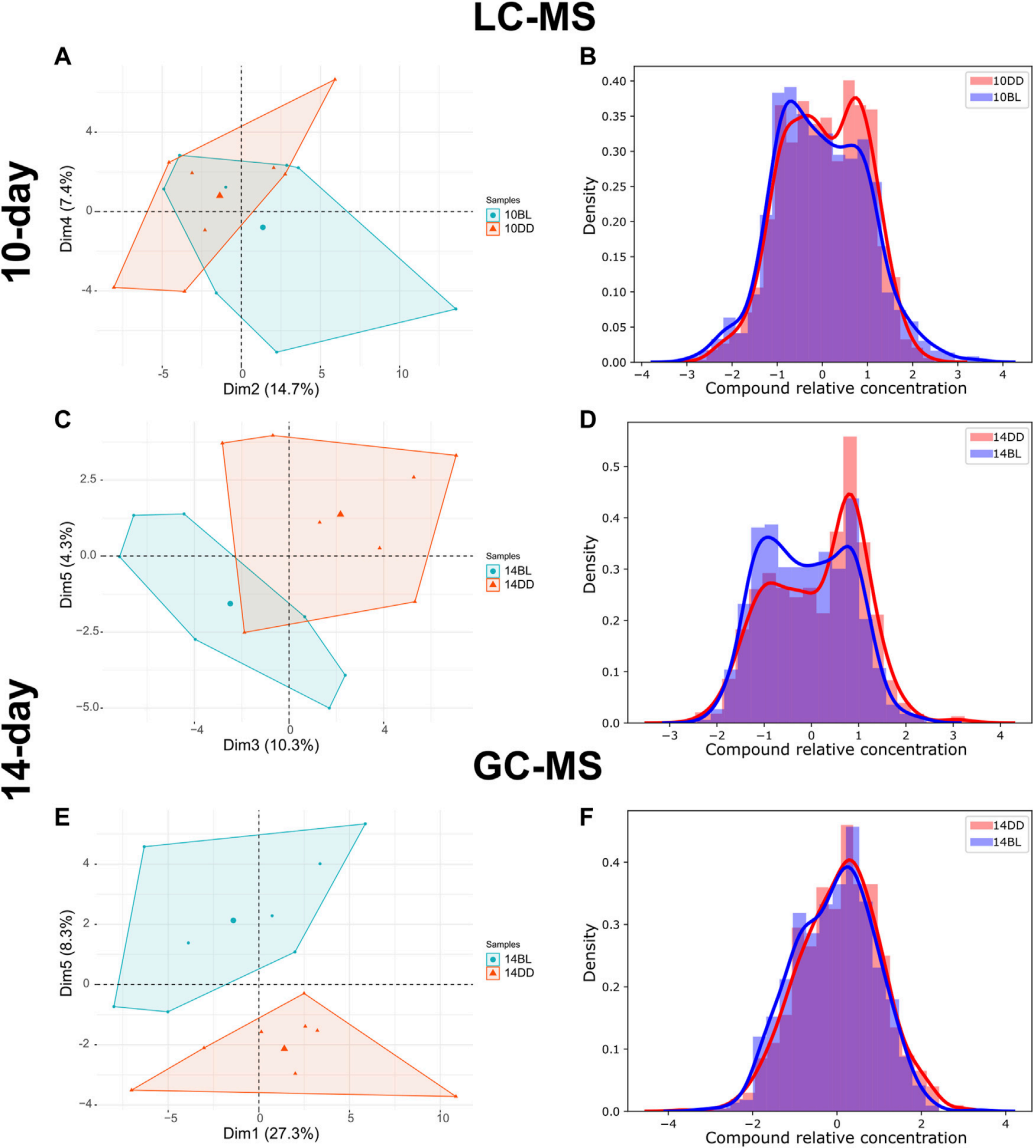
LC- and GCMS detected significant metabolome changes in heads of *eya* ^*2*^ flies kept in BL. **(A,C,E)** PCA plots of LC-MS or GC-MS metabolite data comparing flies kept for 10 or 14 days in BL versus DD. For additional data see Supplementary Figures S1–S3. **(B,D,F)** Density plots for the same datasets. The distribution curve was calculated based on the histogram of the relative concentration distribution (*x*-axis). The probability density function for the concentration distribution was used as the *y*-axis.

**FIGURE 3:**
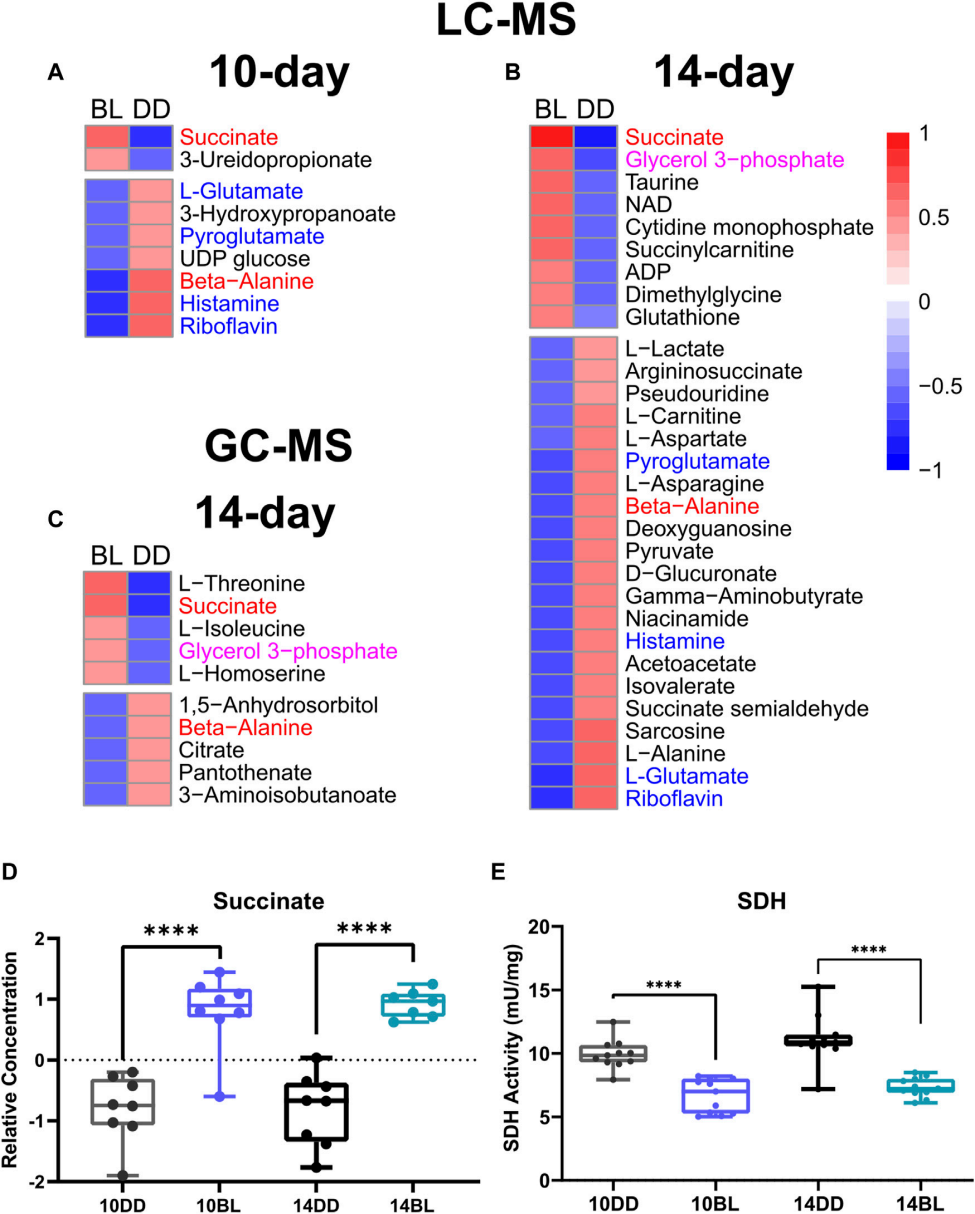
Heat maps showing metabolites significantly altered in response to BL. **(A)** BL exposure for 10 days with detection by LC-MS. **(B)** BL exposure for 14 days with detection by LC-MS. **(C)** BL exposure for 14 days with detection by GC-MS. Labels in red denote metabolites found significant by both methods at both time points, labels in blue show metabolites found significant by LC-MS after both 10 and 14 days of BL exposure, and labels in purple show metabolites found significant after 14 days of BL exposure with both methods. **(D)** Relative concentrations of succinate the most significantly altered metabolite. **(E)** Activity of succinate dehydrogenase (SDH) measured in heads of *eya* ^*2*^ flies was significantly altered after 10 and 14 days of BL exposure. Lines in box plot represent max, upper quartile, median, lower quartile, and min from top to bottom. Statistical analysis by unpaired *t*-test (^∗∗∗∗^ *p* < 0.0001).

LC-MS analysis of flies kept in BL for 10 days detected 175 metabolites, 9 of which were significantly altered compared to the DD control (*t*-test, *p* ≤ 0.05). Principal component analysis (PCA) of metabolites shows weak separation, with a minimum coverage rate, defined by the area intersection of the irregular polygons containing each metabolite, (17.9%, Supplementary Figure S1) PC2 and PC4 explaining 14.7% and 7.4% of the variability, respectively (Figure 2A). The density plot showing the distribution of relative concentration for both BL and DD indicate that BL exposure reduced the level of most metabolites. (Figure 2B). Heatmap shows two metabolites upregulated and seven downregulated by BL (Figure 3A).

LC-MS analysis of flies kept in BL for 14 days detected 176 metabolites, 30 of which were significantly altered in BL flies compared to DD control (*t*-test, *p* ≤ 0.05) demonstrating that changes in metabolites become more dramatic with longer exposure to BL. PCA for this data set is shown in Figure 2C, with a lower minimum coverage rate (4.8%, Supplementary Figure S2) on PC3 and PC5 explaining 10.3% and 4.3% of the variability, respectively. The density plot shows the BL distribution curve shift further to the left, which indicates the metabolome changes caused by BL were more pronounced compared to 10-day BL exposure (Figure 2D). The heatmap shows 9 metabolites upregulated and 21 downregulated in flies kept in BL for 14 days (Figure 3B). Most metabolites altered after 10 days of BL are still detected as significant at 14 days except for uridine diphosphate glucose (UDP-glucose), 3-ureidopropanoate, and hydroxypropionate (*p* = 0.057 in 14-day). Succinate and riboflavin continue to be the most altered metabolites with fold-change of 2.05 increase or 2.17 decrease, respectively.

LC-MS and GC-MS provide both overlapping and distinct capabilities for metabolomics in terms of metabolite detection (Patti et al., 2012; Fiehn, 2016). Therefore, to complement our LC-MS approach, we also used GC-MS to profile the presence and relative abundance of low molecular weight metabolites in BL-treated flies. For these experiments we used flies exposed to BL for 14 days because of more dramatic differences in metabolites detected by LC-MS at this age (Figures 3A,B). GC-MS analysis detected 87 metabolites, 10 of which were significantly changed with BL exposure (*t*-test, *p* ≤ 0.05). Using PCA, metabolome profiles effectively separated BL effects, with PC1 and PC5 explaining 27.3% and 8.3% (Figure 2E). The BL distribution curve was shifted to the left compared with DD control in the density plot, confirming that BL exposure reduced most metabolite levels (Figure 2F). Heatmap shows five upregulated and five downregulated metabolites (Figure 3C). Significant differences in the abundance of succinate, glycerol 3-phosphate (G3P) and beta-alanine were detected by both LC- and GC-MS; however, several significantly altered metabolites were only detected by GC-MS, namely, threonine, isoleucine, homoserine, citrate, and 3-aminoisobutanoic acid, a protective factor against metabolic disorder (Roberts et al., 2014).

Succinate is one of the most significantly increased metabolites (*p* = 8.85 × 10^−05^) detected by both LC- and GC-MS in flies kept in BL for 10 and 14 days (Figure 3D). Increased succinate level suggest that the enzymatic activity of succinate dehydrogenase (SDH) may be compromised; therefore, we measured the activity of SDH in fly heads kept for 10 and 14 days in constant BL and age-matched DD controls. SDH activity was reduced significantly (*p* < 0.0001) in flies exposed to BL for both 10 and 14 days (Figure 3E) indicating that it is an early indicator of metabolic impairment. It appears that SDH deficiency is likely responsible for considerable succinate accumulation, although we note that fumarate, the product of this reaction is not significantly different (Supplementary Table S1).

### Several metabolic pathways are altered in response to constant blue light

To obtain insights into processes most significantly altered by BL exposure, we used MetaboAnalyst 5.0 “Pathway Analysis” function (Pang et al., 2021). We performed this analysis on combined data obtained by LC-MS and GC-MS in flies exposed to BL for 14 days and DD controls. Detailed results of pathway analysis are shown in Supplementary Table S2.

**FIGURE 4:**
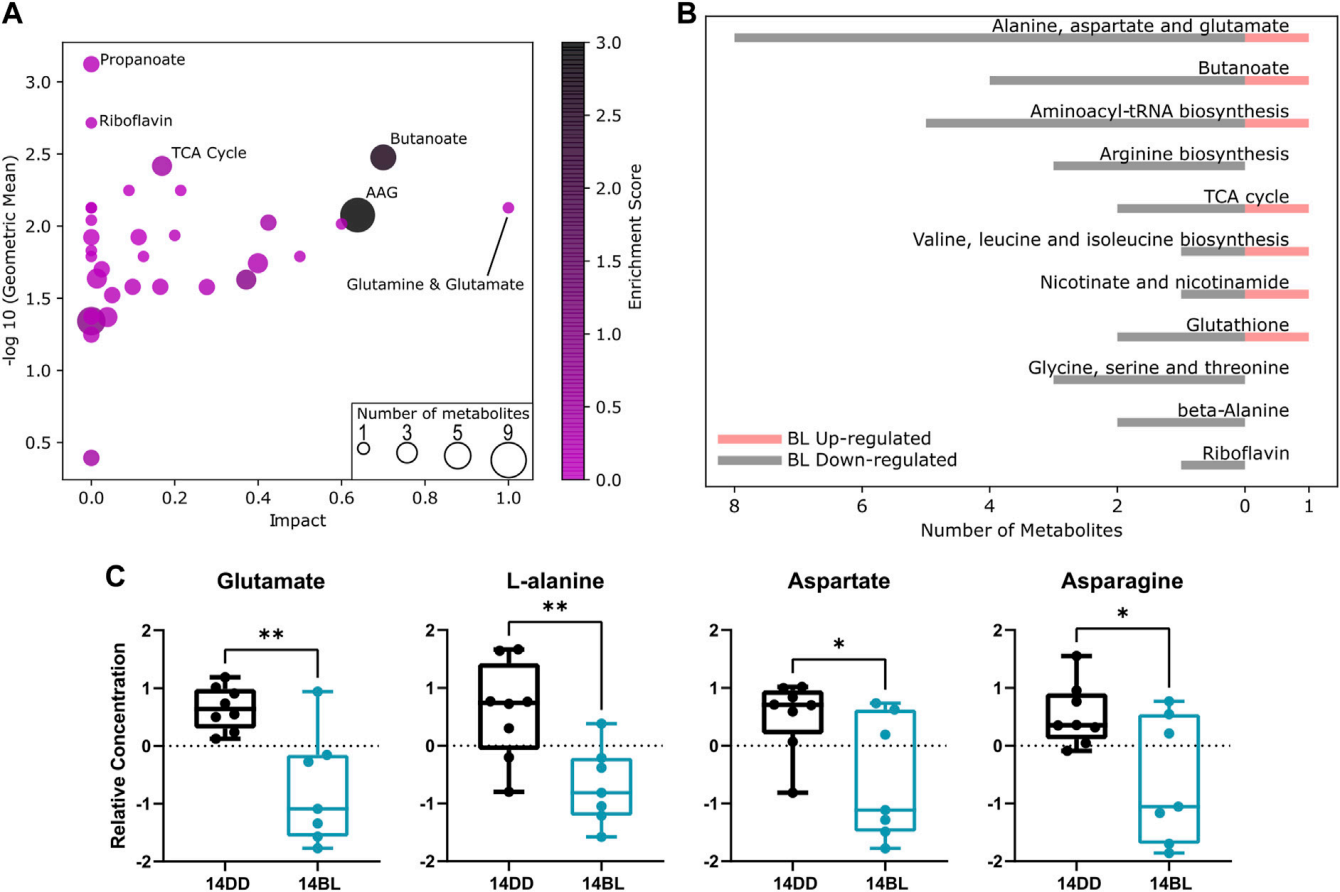
Pathway analysis indicate several processes involving non-essential amino acids are affected by BL. **(A)** Pathway enrichment statistics plot. Each node reflects a significantly altered cluster of metabolites. Pathway impact values from pathway topology analysis are on the *x*-axis. Geometric differences in concentration level between BL exposed and control flies are on the *y*-axis. The node color is based on its enrichment score and the node size is based on the number of significantly altered metabolites in the corresponding pathways. AAG: alanine, aspartate, and glutamate metabolism. **(B)** Histogram representing the top enriched metabolic processes showing number of metabolites up- or downregulated after 14 days of BL. A *p*-value cutoff of 0.1 was used. **(C)** Relative levels of glutamate, L-alanine, aspartate, and asparagine are significantly reduced after 14 days of BL exposure. Lines in box plot represent max, upper quartile, median, lower quartile, and min from top to bottom. Statistical analysis by unpaired *t*-test (^∗∗^ *p* < 0.01, ^∗^ *p* < 0.05).

Pathway enrichment analysis revealed that the propanoate metabolism, tricarboxylic acid (TCA) cycle, riboflavin metabolism, butanoate metabolism, as well as alanine, aspartate and glutamate (AAG) metabolism are the most altered metabolic pathways associated with BL exposure (Figure 4A). Pathways with a *p*-value <0.1 are listed in Figure 4B with the number of metabolites up or downregulated by BL. We note significant reduction in the level of non-essential amino acids including glutamate, alanine, asparagine, and aspartate (Figure 4C), as well as arginosuccinate (Figure 3B), which participate in a number of pathways impacted by BL exposure (AAG, butanoate, arginine biosynthesis).

### Blue light impairs pathways associated with energy metabolism

**FIGURE 5:**
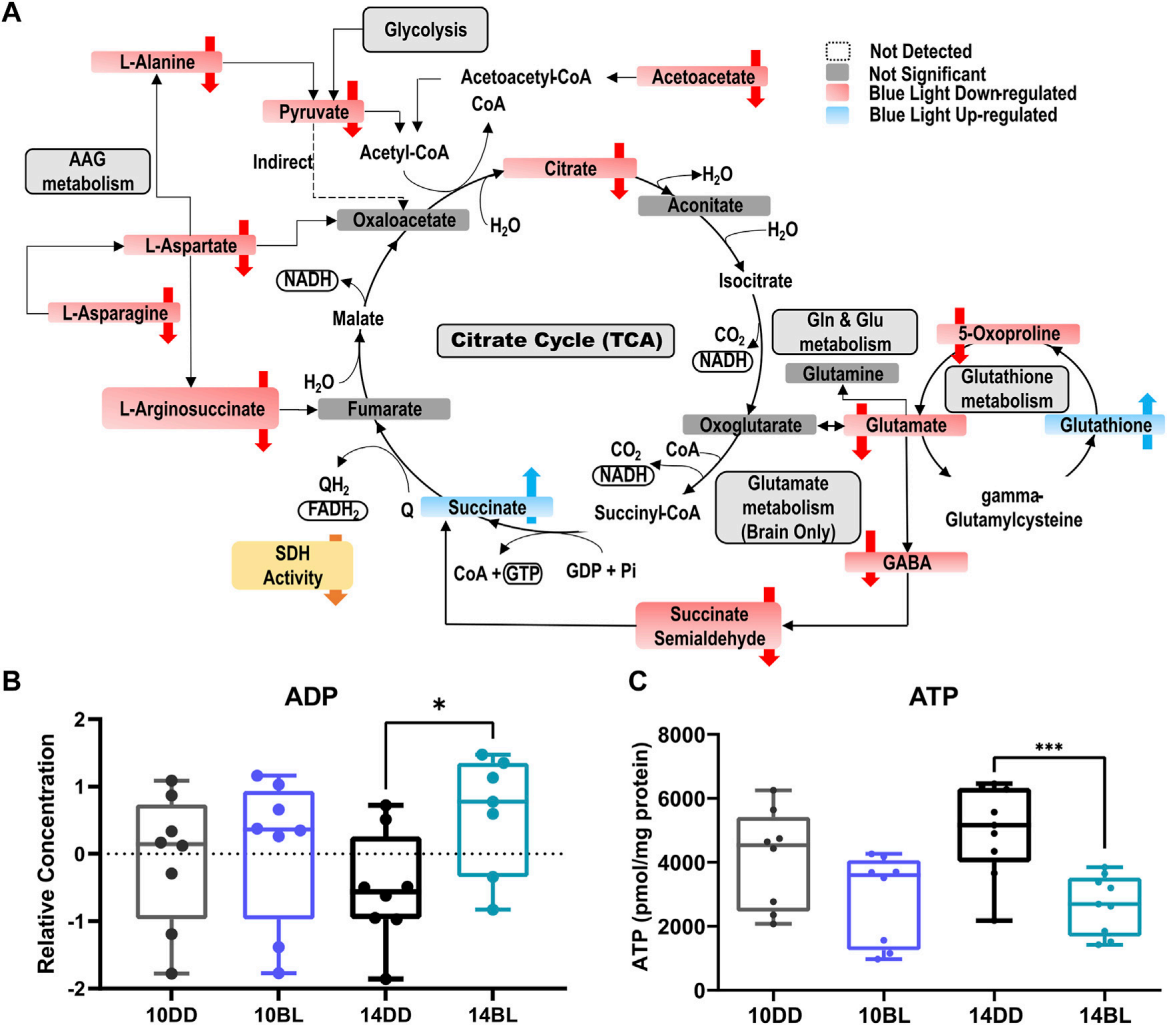
Schematic overview of the metabolic pathways most affected by chronic BL in heads of *eya* ^*2*^ flies. **(A)** Downregulated metabolites are shown in red, and upregulated metabolites in blue. **(B)** Box plots for ADP levels after 10 or 14 days of chronic BL. **(C)** ATP levels in heads of flies after 10 or 14 days of BL. *N* = 6 independent biological replicates of 25 flies for each condition. Lines in box plot represent max, upper quartile, median, lower quartile, and min from top to bottom. Statistical analysis by unpaired *t*-test (^∗∗∗^ *p* < 0.001, ^∗^ *p* < 0.05).

As noted above, succinate is highly elevated in BL-exposed flies possible due to decreased activity of SDH which plays important roles in both the TCA and ETC cycle. In contrast, the levels of several other metabolites feeding into the TCA cycle are significantly reduced including glycolysis-derived pyruvate, acetoacetate, and citrate (Figure 5A). These changes suggest that energy production may be severely impaired in constant BL. In support of this prediction, levels of ADP were significantly increased in BL-exposed flies (Figure 5B). Since ATP was not detected with LC- or GC-MS, we measured steady-state levels of ATP in BL-exposed flies and DD controls using bioluminescent assays. ATP levels were somewhat lower in flies kept for 10 days in BL and highly significantly reduced (*p* < 0.001) after 14 days in BL compared to DD controls (Figure 5C). It should be noted that our analysis detected elevated levels of G3P (Figures 3B,C), which allows the NADH synthesized in the cytosol by glycolysis to contribute to the oxidative phosphorylation pathway in the mitochondria to generate ATP.

### Blue light exposure alters levels of several brain neurotransmitters

**FIGURE 6:**
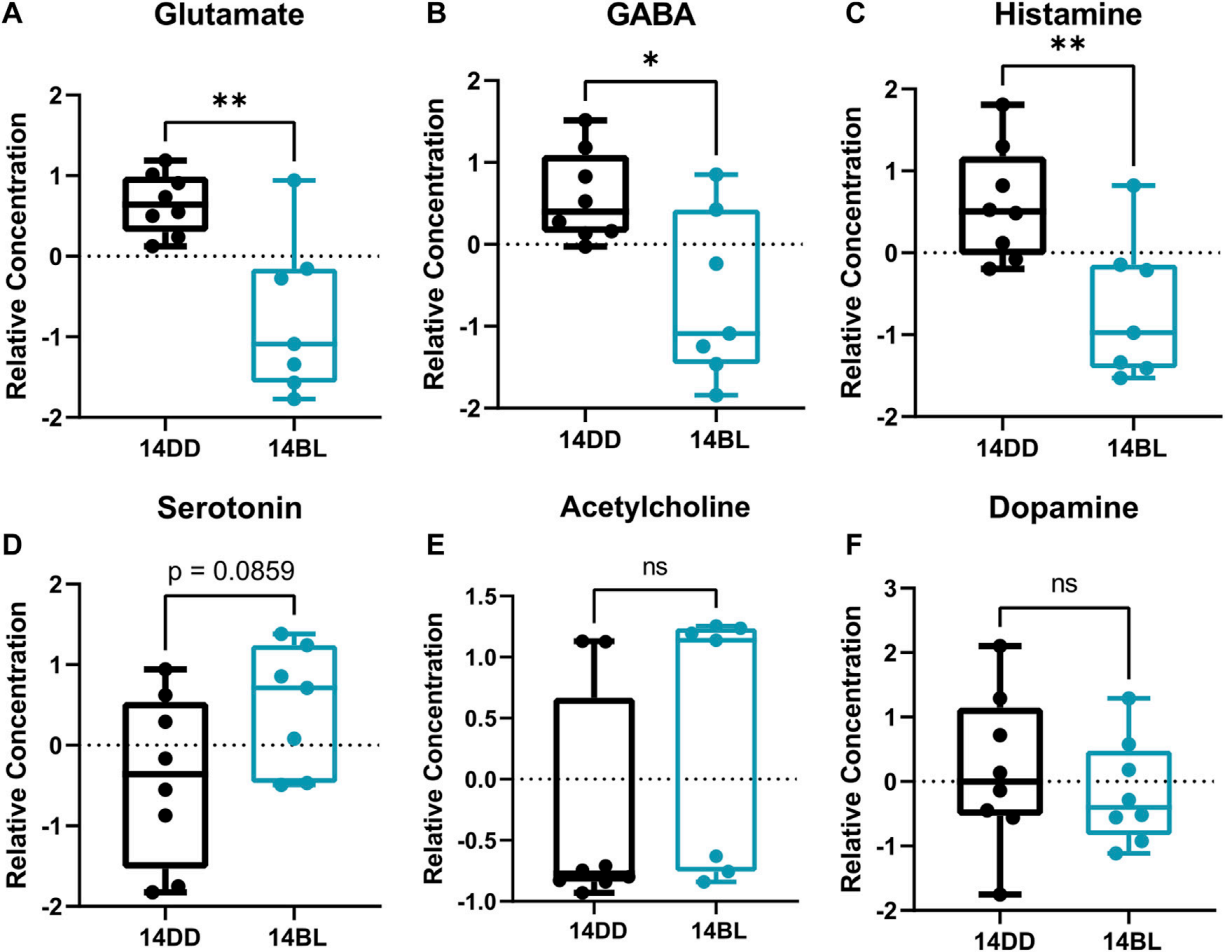
Some neurotransmitters are significantly reduced after 14 days of chronic BL. **(A–F)** Relative concentration of Glutamate, GABA, and Histamine are significantly lower after 14 days of chronic BL but others are not altered. Lines in box plot represent max, upper quartile, median, lower quartile, and min from top to bottom. Statistical analysis by unpaired *t*-test (^∗∗^ *p* < 0.01, ^∗^ *p* < 0.05, ns *p* > 0.05).

Given that flies exposed to BL for 14 days showed significant brain neurodegeneration (Figure 1D), we sought to identify any changes in neurotransmitters and neuromodulators in these flies. Our data show that the levels of both, excitatory glutamate and inhibitory GABA neurotransmitters are significantly reduced (Figures 6A,B). In addition, histamine levels are significantly reduced (Figure 6C); while histamine is a well-known neurotransmitter in the fly photoreceptors (Borycz et al., 2012), detecting it in eyeless flies is consistent with a recent report that histamine signaling functions in other parts of the brain where it is involved in modulating temperature preference in flies (Hong et al., 2006). Interestingly, histamine is inactivated by conjugation to β-alanine (Borycz et al., 2012), which is also significantly decreased in heads of flies kept in BL for 14 days (Figures 3B,C). The levels of acetylcholine and dopamine were not different between BL and DD flies (Figures 6E,F), while serotonin was modestly elevated in brains of BL flies (Figure 6D). Taken together, these data suggest that BL leads to an imbalance in the levels of neurotransmitters in eyeless flies.

### Supplementation of reduced metabolites does not extend lifespan in blue light-exposed flies.

Several of the metabolites that were significantly reduced in BL-exposed flies play important roles in metabolic homeostasis. One such metabolite is glutamate, which is at the crossroad between multiple metabolic pathways (Figure 5A). To test if glutamate deficiency is causative in accelerated aging in BL-exposed flies, we supplemented the fly diet with 200 and 400 μg/ml of glutamate and recorded their mortality. We found that 200 μg/ml glutamate did not significantly alter lifespan of flies kept in constant BL, while 400 μg/ml shortened lifespan compared to no glutamate control (Supplementary Figure S4).

Our data revealed that riboflavin (vitamin B2) was the most significantly reduced metabolite (*p* = 4.08 × 10^−04^) in BL-exposed flies (Supplementary Table S1, Supplementary Figure S5A). Given that riboflavin is a precursor to FMN and FAD, which serve as cofactors in many proteins involved in energy metabolism, we tested the effects of riboflavin supplementation on lifespan of *eya* ^*2*^ flies in chronic BL. Fly diet was enriched with 200 μg/ml and 400 μg/ml of riboflavin and mortality was recorded. As shown in Supplementary Figure S5B,C, both concentrations shortened the lifespan compared to the no riboflavin control.

## Discussion

Natural light is necessary for life, but prolonged exposure to artificial light with a high content of blue wavelengths, is a matter of increasing concern for human health, especially with regard to retinal pathologies. However, recent data on the model organisms *C. elegans* (De Magalhaes Filho et al., 2018) and *D. melanogaster* (Nash et al., 2019; Song et al., 2022) demonstrate that cells and tissues not specialized for light perception, such as brain, can be damaged in flies kept in BL for extended time. In the present study, we investigated effects of chronic BL on metabolic pathways in heads of flies with genetically ablated eyes in order to focus on extra-retinal tissues. Our metabolomic analyses revealed alterations in the levels of several metabolites suggesting that energy production and other cellular pathways are significantly altered in these flies. In particular, dramatic metabolic rearrangements are observed in heads of flies kept in BL for 14 days. These flies show onset of neurodegeneration, consistent with accelerated aging; yet, their lifespan can be rescued if they are returned to DD. Thus, our results may provide insights into both mechnisms that initially protect fly physiology, and metabolic impairments that could damage fly cells upon longer BL exposure.

In our previous work, we showed that damaging effects of blue light significantly increase with the age at which flies are exposed to this stressor. We reported that blue light reduces the activity of Complex II in the electron transport system in both young and old flies. In addition, complex I and complex IV activity are reduced with aging, independent of light exposure (Song et al., 2022). To avoid confounding effects of age-associated metabolomic changes while investigating the impact of BL on the metabolome, the experiments reported here were conducted on young flies exposed to BL for increasing number of days: 10, 14, and 16 days. In this way, our data pertain to specific aspects of cellular damage by BL, irrespective of chronological age.

Metabolic profile analysis shows that succinate is the most significantly increased metabolite in flies kept in BL for both 10 and 14 days. This increase may be caused, at least in part, by reduced activity of succinate dehydrogenase (SDH), which catalyzes succinate oxidation to fumarate and transfers electrons from succinate to ubiquinone *via* complex II in the mitochondrial electron transport chain. These data support our previous report on mitochondria respiration showing that blue light specifically reduces the activity of Complex II (Song et al., 2022). A previous study investigating impaired SDH assembly and activity in *Drosophila* reported elevated succinate levels and also reduced fumarate (Van Vranken et al., 2014). We did not detect a decrease in fumarate, which could possibly be supplied by degradation of tyrosine or other amino acids.

In addition to acting as an essential intermediate of the TCA cycle, succinate exerts pleiotropic roles beyond metabolism in both physiological and pathological conditions. Succinate produced in mitochondria may be transported to the cytosol and promote protein succinylation, a post-translational modification with the potential to impact protein function (Grimolizzi and Arranz, 2018). Further, succinate is emerging as a systemic metabolic signal that activates specific receptors (conserved in flies) and can affect gene expression to modulate energy metabolism (Murphy and Chouchani, 2022). In glioma and other cancer cells, a decrease or loss of SDH activity leads to accumulation of succinate concomitant with reduced levels of citrate and several non-essential amino acids: glutamate, alanine, aspartate, and asparagine (Lussey-Lepoutre et al., 2015). Remarkably, we observed a similar metabolic phenotype with reduced levels of the same compounds in heads of flies exposed to BL, suggesting, that BL-induced impairment of SDH may trigger metabolic rearrangements induced by elevated succinate.

Our pathway enrichment analysis showed that the TCA cycle is significantly impacted by BL. TCA is a central metabolic pathway responsible for supplying reducing potential for oxidative phosphorylation and anabolic substrates for cell growth, repair, and proliferation. In contrast to elevated succinate, other metabolites feeding into the TCA cycle, pyruvate and citrate, were significantly reduced (Figure 5). Also decreased were acetoacetate, L-aspartate, B-alanine, and sarcosine, which can be converted to acetyl-CoA by different enzymes. While acetyl-CoA was not detected in our study, our data provide solid evidence that the TCA cycle is dysregulated in BL leading to decreased energy production. Indeed, we detected elevated levels of ADP by LC-MS and reduced levels of ATP (*via* bioluminescence assay) in heads of flies exposed to BL for 14 days. It is likely that ATP deficiency becomes more aggravated with longer BL exposure, leading to increased neurodegeneration followed by death in these conditions.

One of the important findings in our metabolite analysis is the apparent imbalance in the levels of neurotransmitters in brains of flies held in BL for 14 days, which showed significant brain neurodegeneration. We found that histamine levels were significantly decreased, which was surprising given that histamine is a well-known neurotransmitter in the fly photoreceptors (Borycz et al., 2012). However, histamine is present in other parts of the brain and it is involved in modulating locomotor behavior (Hong et al., 2006). In addition, levels of the amino acid glutamate which acts as excitatory neurotransmitter are significantly reduced. Glutamate is a precursor neurotransmitter GABA, which is also significantly reduced in BL-exposed flies. Since the levels of other neuromodulators are not affected, it appears that glutamate deficiency may be the culprit for the observed changes.

Glutamate participates in several metabolic pathways that showed significant enrichment in our analysis including glutathione (GSH) synthesis (Figure 5). Remarkably, GSH is significantly elevated in BL-exposed flies. GSH plays critical roles in protecting cells from oxidative damage and maintaining redox homeostasis. It has been reported that transcription and activity of GSH-producing enzymes increase in aged flies (Klichko et al., 2015; Sebastian et al., 2022); therefore, it is tempting to speculate that glutamate may be diverted into GSH synthesis away from its other roles. However, we found that glutamate supplementation did not increase lifespan under blue light.

One of the most depleted metabolites in both 10- and 14-day BL flies is riboflavin (Supplementary Figure S5A). Riboflavin is a precursor to flavin adenine mononucleotide (FMN) and flavin adenine dinucleotide (FAD), an essential cofactor in many redox enzymes including SDHA, the catalytic subunit of the SDH complex. Riboflavin is maximally activated by BL of 450 nm, close to the peak of 460 nm, which was used in our study. BL can degrade riboflavin into non-active photoproducts (Sheraz et al., 2014), yet BL does not significantly alter FAD levels in our study suggesting that FAD in flavinated proteins may be somehow protected from BL. Interestingly, it was recently reported that riboflavin is one of the most depleted metabolites in the aging *Drosophila* eye in regular light/dark cycles (Hall et al., 2021) and in another study (Zou et al., 2017), riboflavin supplementation extended fly lifespan. We tested lifespan of *eya* ^*2*^ flies in chronic blue light on diet supplemented with riboflavin and found that the lifespan was shorter rather than extended, suggesting a more complex regulatory role of riboflavin in the presence of blue light.

We recognize that there are some limitations to our study. First, although several brain-specific neuromodulators were captured in our study, we analyzed aggregate metabolites present in whole head (albeit without compound eyes), which include not only brains, but also fat body, muscle and epithelial cells. Second, we did not measure changes in lipid metabolites, therefore, contribution of lipids to oxidative phosphorylation in BL-exposed flies remains unknown. Finally, steady-state levels of metabolites do not provide information on how metabolic flux is altered by BL exposure and future research need to address this question.

In summary, our metabolomic results provide novel insights into the mechanisms by which BL interferes with vital metabolic pathways in extra-retinal cells in flies. All metabolites altered by BL in our study are conserved between fly and human cells. Therefore, it is possible that prolonged exposure to BL may have similar, albeit more subtle effects on skin, subcutaneous fat, and other cells in the human.

## Data Availability

The original contributions presented in the study are included in the article/Supplementary Material, further inquiries can be directed to the corresponding authors.
