# Supplementary figures and images for: Chronic blue light leads to accelerated aging in Drosophila by impairing energy metabolism and neurotransmitter levels
Source: Front Aging. 2022 Aug 31;3:983373. doi: 10.3389/fragi.2022.983373 (PMC9479496; doi:10.3389/fragi.2022.983373)

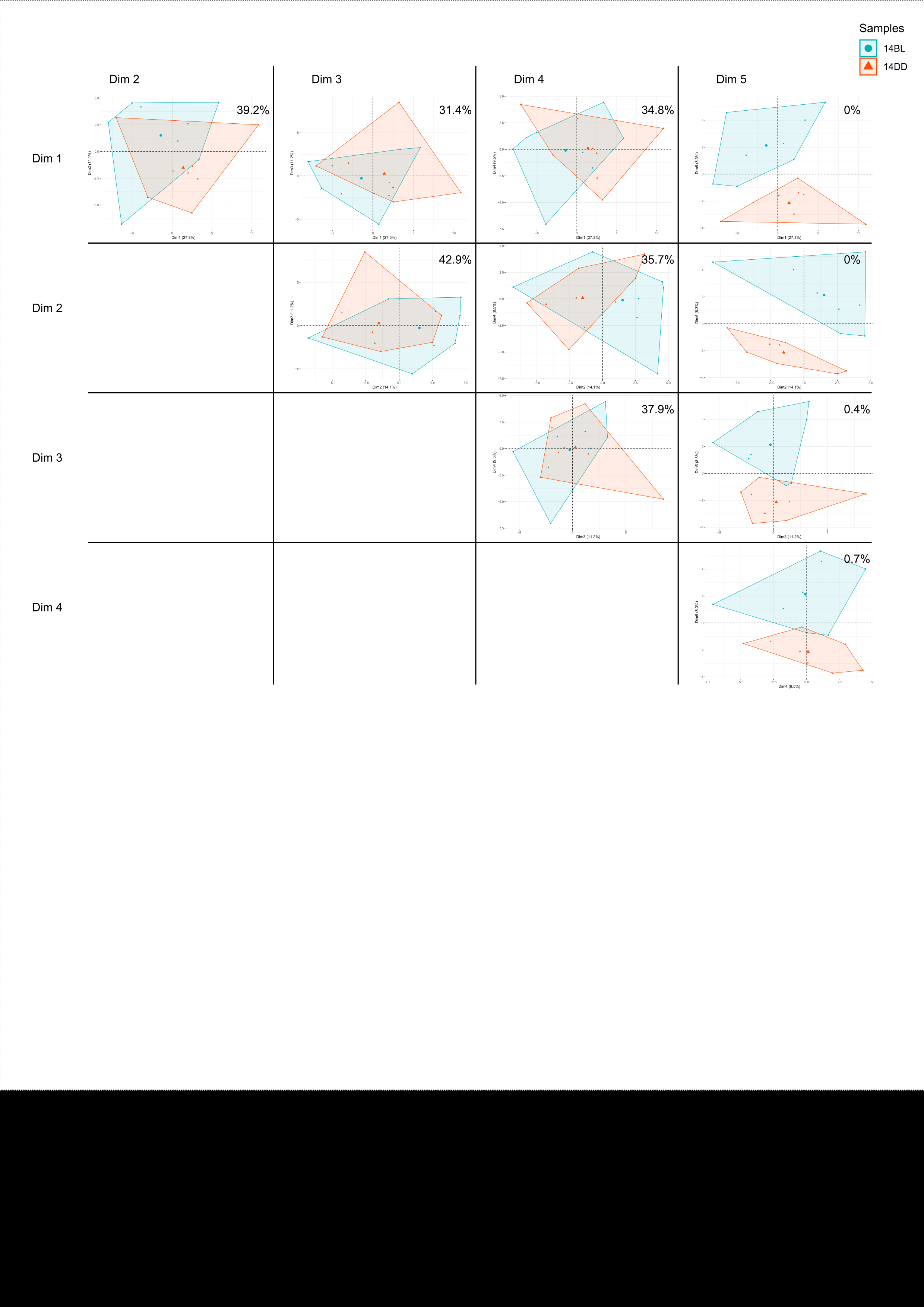

Supplement: Supplementary file 1 [file Image3.tiff]

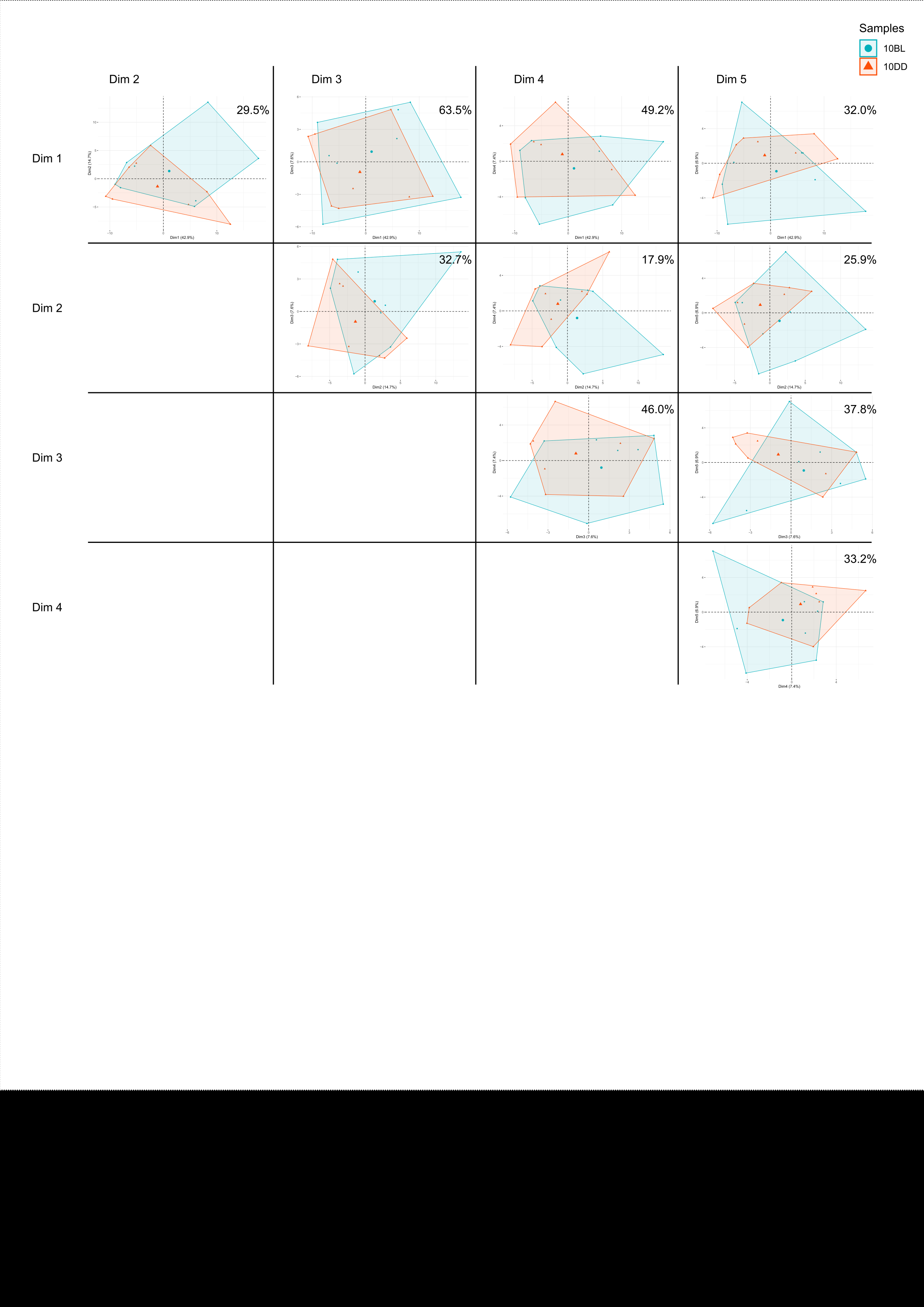

Supplement: Supplementary file 3 [file Image1.tiff]

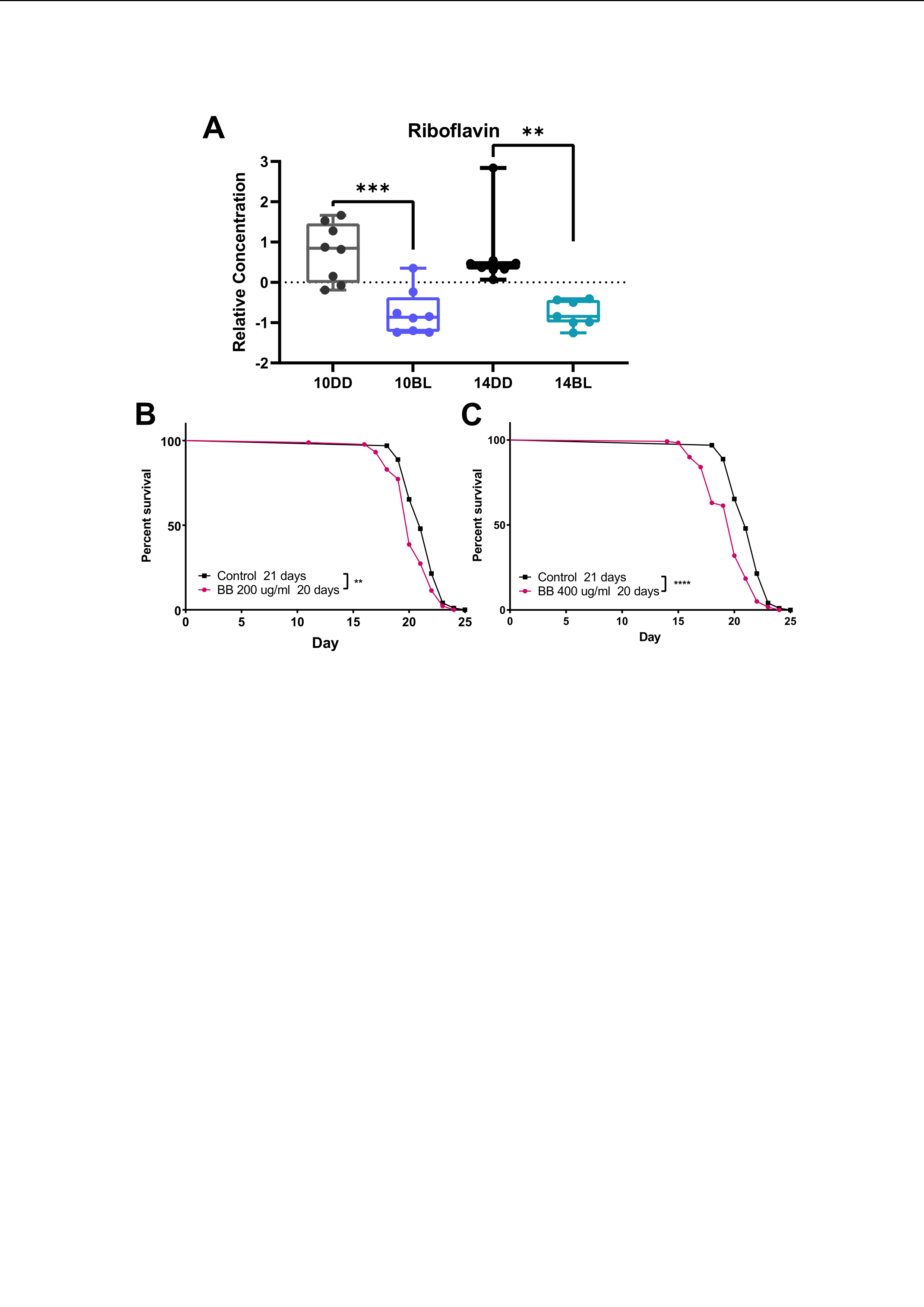

Supplement: Supplementary file 4 [file Image5.tiff]

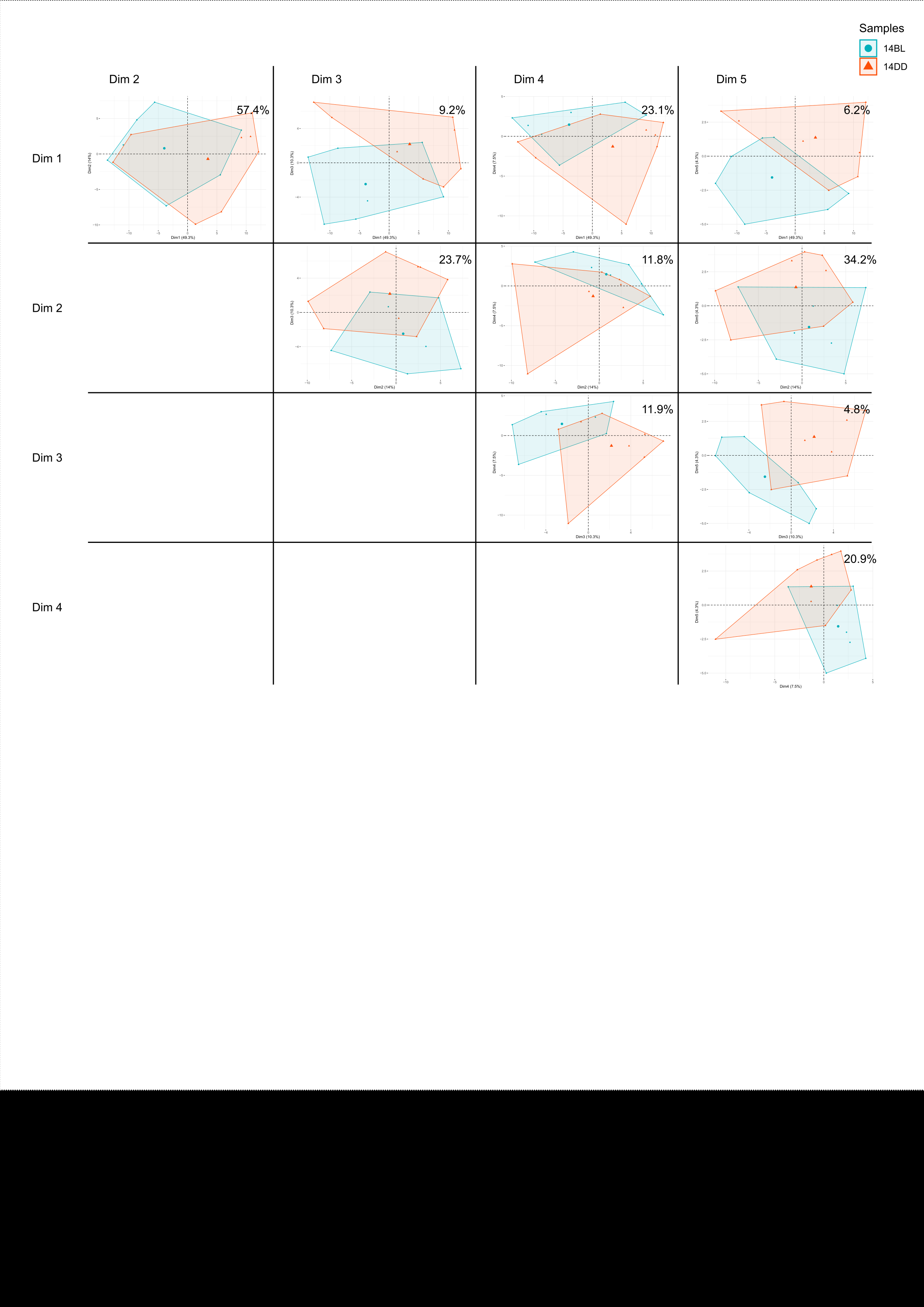

Supplement: Supplementary file 6 [file Image2.tiff]

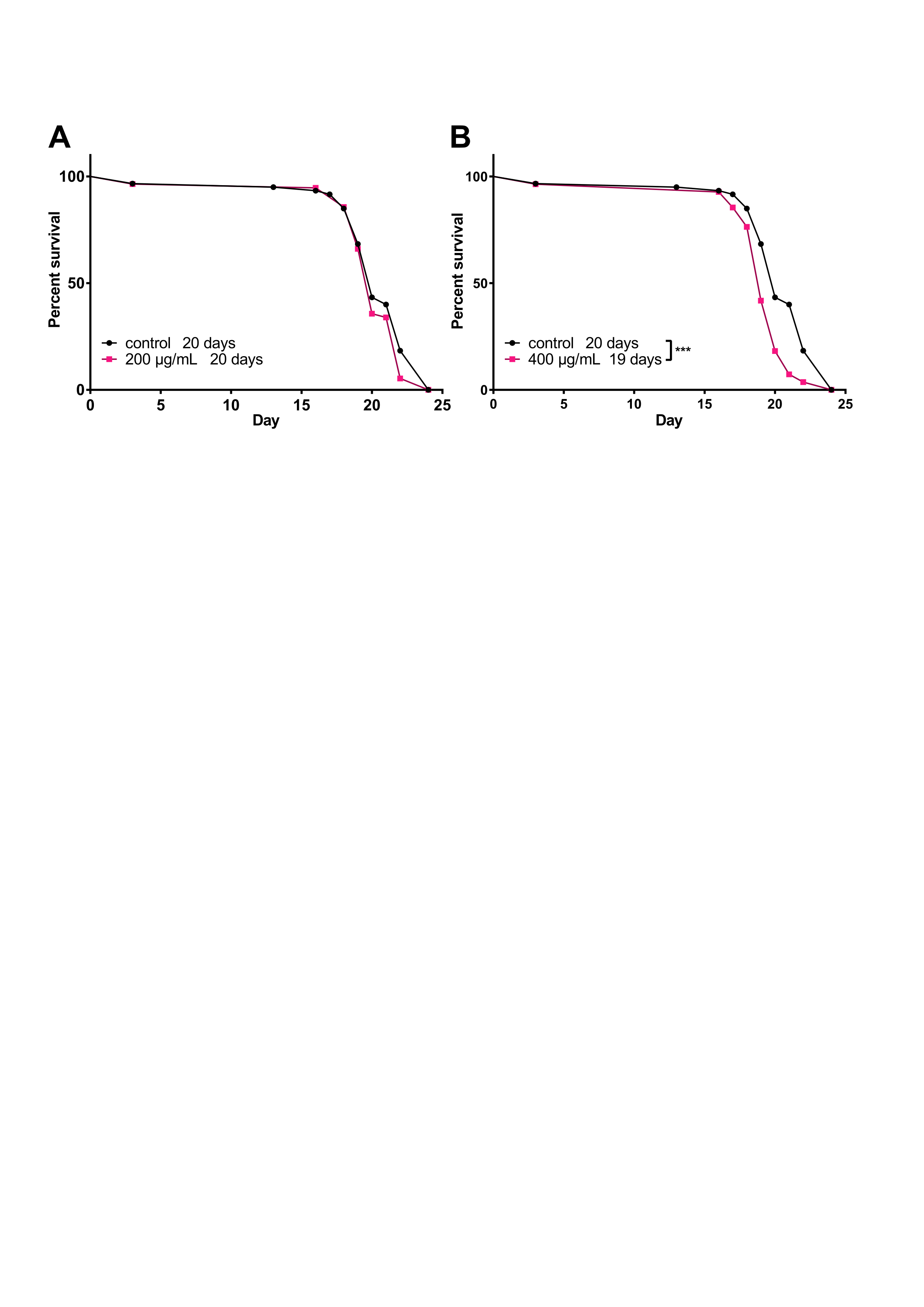

Supplement: Supplementary file 7 [file Image4.tiff]
