# Supplementary material for: Chronic blue light leads to accelerated aging in Drosophila by impairing energy metabolism and neurotransmitter levels
Source: Front Aging. 2022 Aug 31;3:983373. doi: 10.3389/fragi.2022.983373 (PMC9479496; doi:10.3389/fragi.2022.983373)
Supplement: Supplementary file 8 [file DataSheet1.DOCX]

**Chronic blue light leads to accelerated aging in Drosophila by impairing energy metabolism and neurotransmitter levels.**

Yang et al

**SUPPLEMENTARY MATERIAL**

**SUPPLEMENTARY TABLES**

**Supplemental Table 1. Summary of metabolite changes detected with LC-MS and GC-MS at 10- and 14-days.** 214 metabolites are shown in this table with t-stat, p-value, and low or high in BL corresponding to the days. Metabolites detected with both methods are shown with yellow background, those only detected by LC-MS are shown with clear background, and only detected by GCMS are shown with grey background. P value of corresponding metabolite is shown in the table if p-value < 0.1 (red p<0.02, green 0.02<p<0.05, blue 0.05<p<0.1).

**Supplemental Table 2. Summary of pathway analysis.** Table includes all pathways identified by Metaboanalyst 5.0 web server. Metabolites related to each pathway were listed at the end of each line.

**SUPPLEMENTARY FIGURES**

Supplementary Fig 1. PCA analyses in d10 LC-MS metabolomes, including the overlap ratio of each PCA plot for the first five PCs.

Supplementary Fig 2. PCA analyses in d14 LC-MS metabolomes, including the overlap ratio of each PCA plot for the first five PCs.

Supplementary Fig 3. PCA analyses in d14 GC-MS metabolomes, including the overlap ratio of each PCA plot for the first five PCs.

Supplementary Fig 4. Glutamate supplementation did not extend lifespan in flies under BL.

Supplementary Fig 5. Riboflavin supplementation did not extend lifespan in flies under BL.


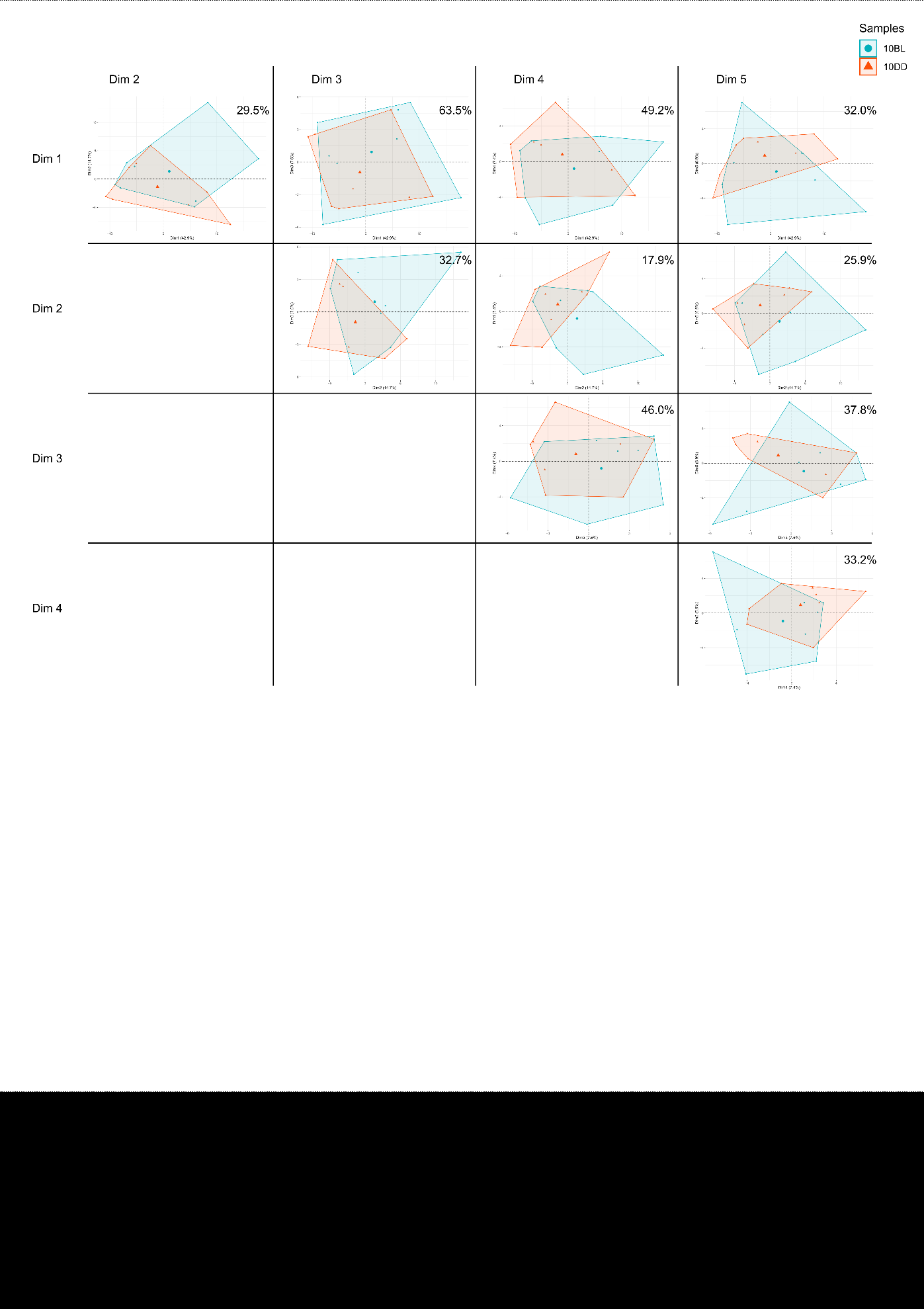


**Fig S1.** **PCA analyses in d10 LC-MS metabolomes, including the overlap ratio of each PCA plot for the first five PCs.**

**
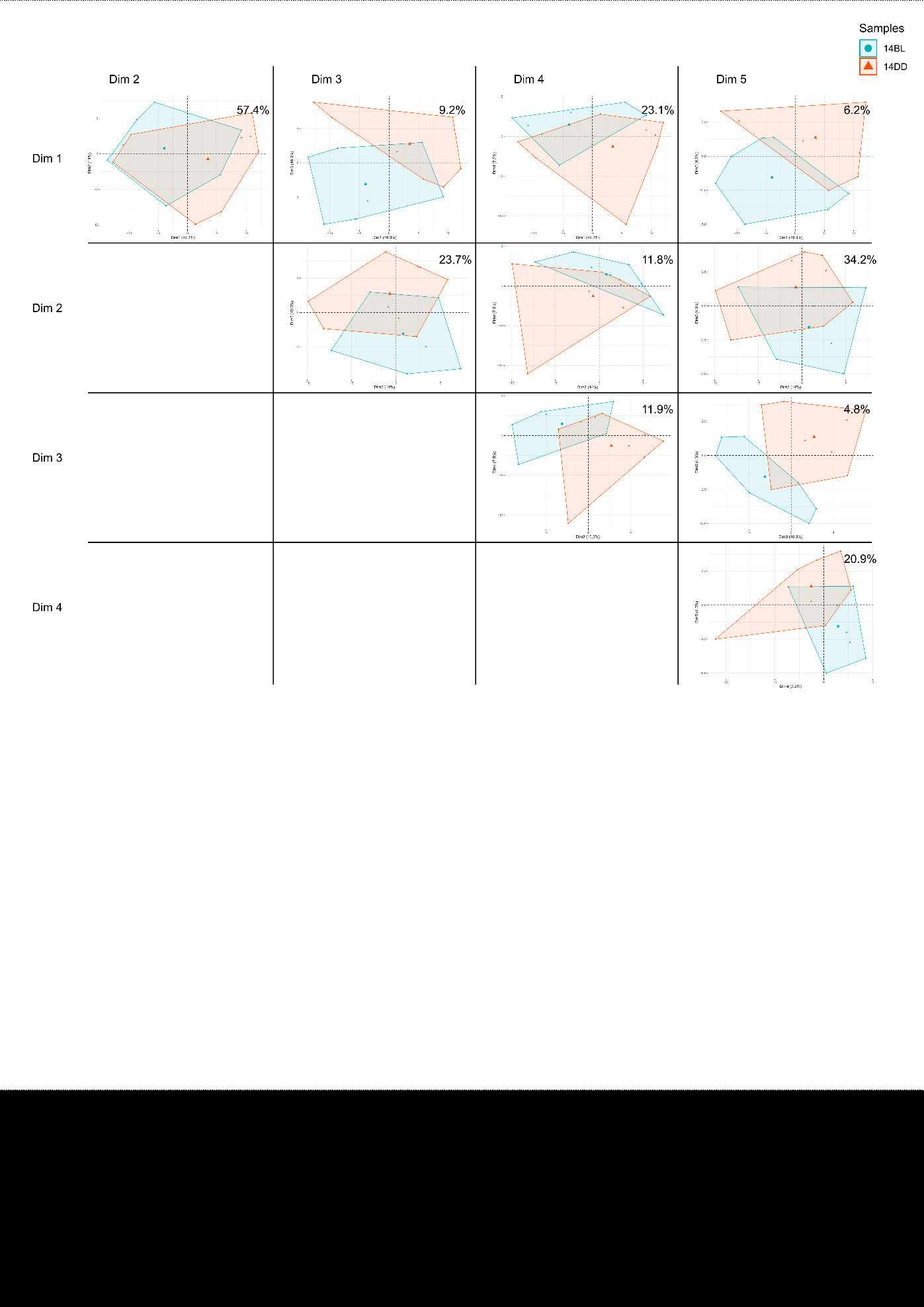
**

**Fig S2. PCA analyses in d14 LC-MS metabolomes, including the overlap ratio of each PCA plot for the first five PCs.**


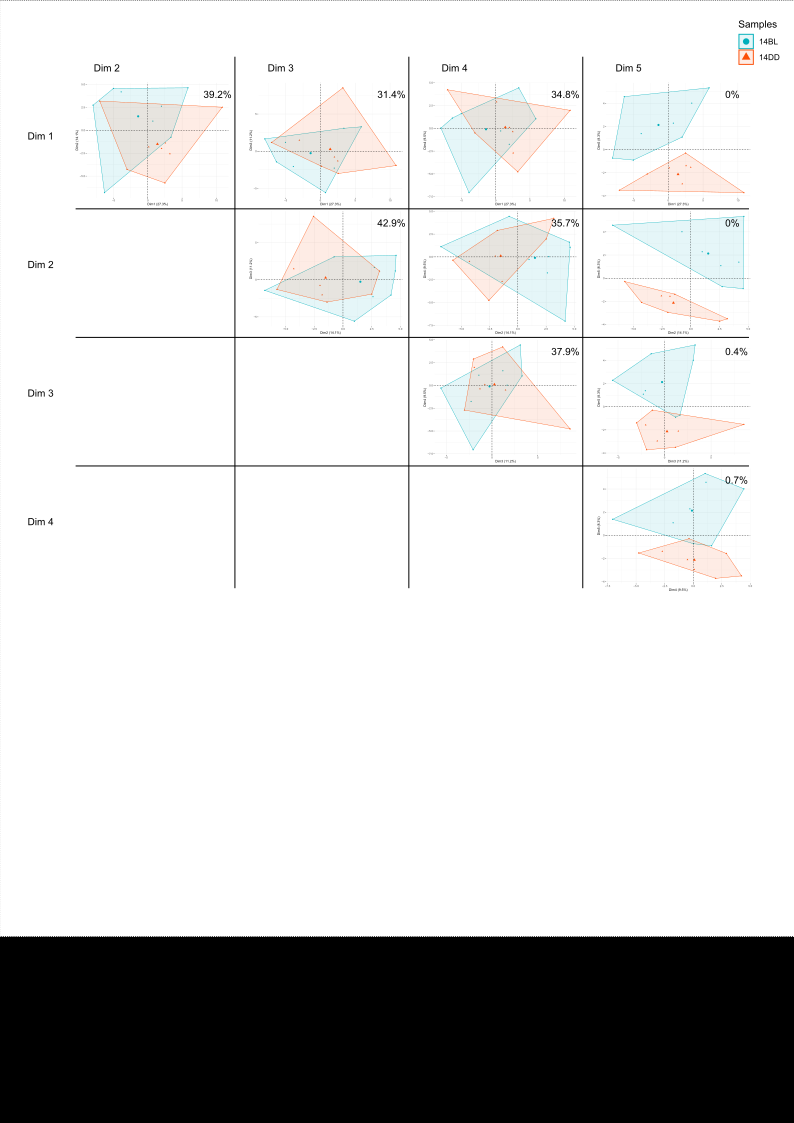


**Fig S3. PCA analyses in d14 GC-MS metabolomes, including the overlap ratio of each PCA plot for the first five PCs.**

**
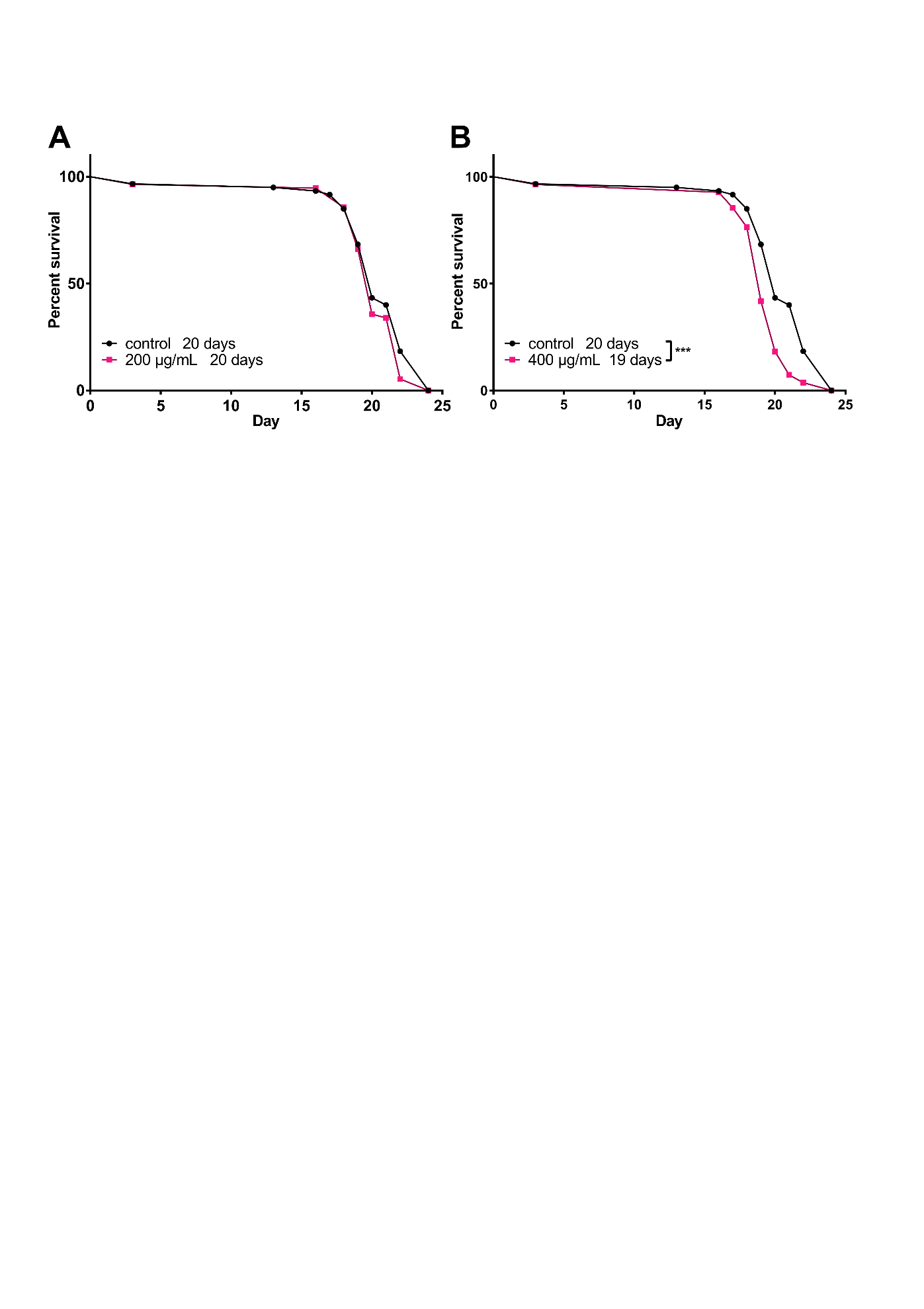
**

**Fig S4. Glutamate supplementation did not extend lifespan in flies under BL.** Lifespan was measured in *eya^2^* flies kept in constant BL with 200 (A) or 400 (B) µg/ml glutamate in the diet. Stars indicate significant differences in survival in BL based on statistics by log-rank test (***p<0.001).


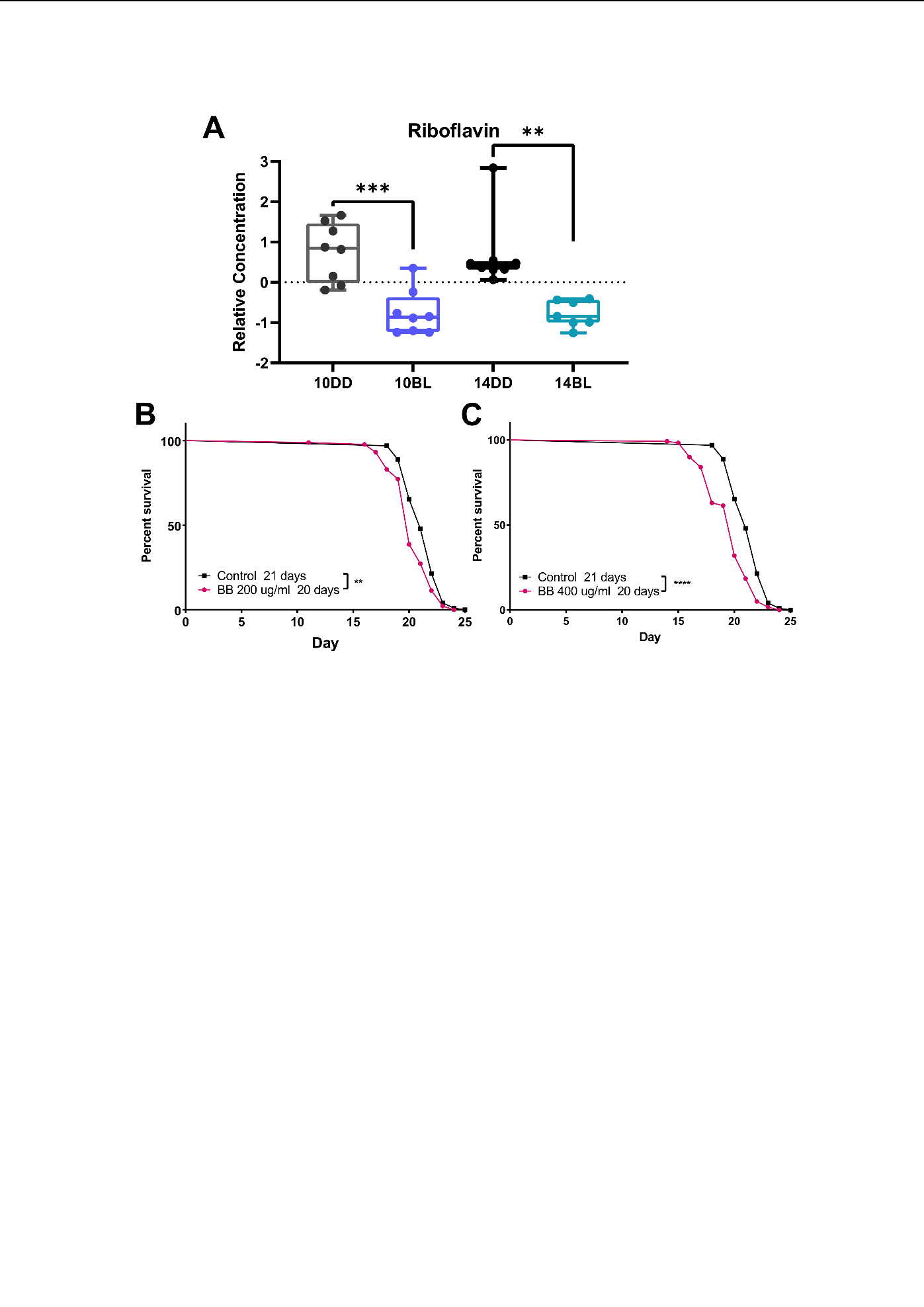


**Fig S5. Riboflavin supplementation did not extend lifespan in flies under BL.** (A) Box plots of riboflavin levels after 10 or 14 days of chronic BL. (B-C) Lifespan was measured in *eya^2^* flies kept in constant BL with 200 (B) or 400 (C) µg/ml riboflavin in the diet. Stars indicate significant differences in survival in BL based on statistics by log-rank test (**p<0.01, ****p<0.0001).
